# Observational Cohort Study of Ventricular Arrhythmia in Adults with Marfan Syndrome Caused by *FBN1* Mutations

**DOI:** 10.1371/journal.pone.0081281

**Published:** 2013-12-13

**Authors:** Ali Aydin, Baran A. Adsay, Sara Sheikhzadeh, Britta Keyser, Meike Rybczynski, Claudia Sondermann, Christian Detter, Daniel Steven, Peter N. Robinson, Jürgen Berger, Jörg Schmidtke, Stefan Blankenberg, Stephan Willems, Yskert von Kodolitsch, Boris A. Hoffmann

**Affiliations:** 1 Department of Cardiology, University Heart Center, University Hospital Eppendorf, Hamburg, Germany; 2 Department of Cardiology/Electrophysiology, University Heart Center, University Hospital Eppendorf, Hamburg, Germany; 3 Department of Medical Biometry and Epidemiology, University Hospital Eppendorf, Hamburg, Germany; 4 Institute of Human Genetics, Hannover Medical School, Charité Universitätsmedizin Berlin, Berlin, Germany; 5 Institute of Human Genetics and Medical Genetics, Charité Universitätsmedizin Berlin, Berlin, Germany; University of Cologne, Germany

## Abstract

**Background:**

Marfan syndrome is associated with ventricular arrhythmia but risk factors including *FBN1* mutation characteristics require elucidation.

**Methods and Results:**

We performed an observational cohort study of 80 consecutive adults (30 men, 50 women aged 42±15 years) with Marfan syndrome caused by *FBN1* mutations. We assessed ventricular arrhythmia on baseline ambulatory electrocardiography as >10 premature ventricular complexes per hour (>10 PVC/h), as ventricular couplets (Couplet), or as non-sustained ventricular tachycardia (nsVT), and during 31±18 months of follow-up as ventricular tachycardia (VT) events (VTE) such as sudden cardiac death (SCD), and sustained ventricular tachycardia (sVT). We identified >10 PVC/h in 28 (35%), Couplet/nsVT in 32 (40%), and VTE in 6 patients (8%), including 3 with SCD (4%). PVC>10/h, Couplet/nsVT, and VTE exhibited increased N-terminal pro–brain natriuretic peptide serum levels(*P*<.001). All arrhythmias related to increased NT-proBNP (*P*<.001), where PVC>10/h and Couplet/nsVT also related to increased indexed end-systolic LV diameters (*P* = .024 and *P* = .020), to moderate mitral valve regurgitation (*P* = .018 and *P* = .003), and to prolonged QTc intervals (*P* = .001 and *P* = .006), respectively. Moreover, VTE related to mutations in exons 24–32 (*P* = .021). Kaplan–Meier analysis corroborated an association of VTE with increased NT-proBNP (*P*<.001) and with mutations in exons 24–32 (*P*<.001).

**Conclusions:**

Marfan syndrome with causative *FBN1* mutations is associated with an increased risk for arrhythmia, and affected persons may require life-long monitoring. Ventricular arrhythmia on electrocardiography, signs of myocardial dysfunction and mutations in exons 24–32 may be risk factors of VTE.

## Introduction

Marfan syndrome is an autosomal-dominantly inherited disease of the connective tissue that is caused by mutations of the fibrillin-1 (*FBN1*) gene, which encodes fibrillin-1 monomers of the extracellular microfibrils [1]. Defective fibrillin-1 may cause Marfan syndrome phenotype by disrupting the structure of the connective tissue elastic fibres [2]. Moreover, defective fibrillin-1 alters transforming growth factor-ß (TGF-ß) signaling, which in *FBN1* deficient mice has been shown to account for manifestations of Marfan syndrome such as pulmonary emphysema, mitral valve prolapse, skeletal muscle myopathy and aortic root dilatation [3]–[6]. In untreated Marfan syndrome dissection and rupture of the proximal aorta are major causes of premature death, but current medical and surgical therapy prevents most of these fatalities [7], [8].

There is mounting evidence, that Marfan syndrome also carries a risk for ventricular arrhythmia and sudden cardiac death (SCD) [9]–[12]. Classically, arrhythmia is viewed not as a primary feature of Marfan syndrome itself, but rather as a result from secondary conditions such as myocardial ischemia, mitral valve abnormality, or ventricular dysfunction [13]. However, the scarce studies of SCD do not use current criteria of Marfan syndrome [9], [10], they do not document presence of causative *FBN1* mutations [9]–[12], and they do not investigate the role of different types of *FBN1* mutations on the risk of ventricular arrhythmia and SCD [9]–[12]. However, such investigations may be important, especially in the light of a recent study of patients with coronary artery disease that documents association of SCD with *TGFBR2* polymorphism and that thereby suggests a pathogenetic link between SCD and altered TGF-ß signaling [14]. Thus, we performed this observational cohort study of adults with *FBN1* gene mutations to elucidate the impact of both clinical characteristics and *FBN1* mutation characteristics on the risk of ventricular arrhythmia and SCD.

## Methods

The Hamburg research ethics committee approved the protocol and approval process. The study was conducted in accordance with the provisions of the Declaration of Helsinki and amendments. All subjects were informed individually and provided their informed consent in writing; next of kin, caretakers, or guardians signed consents on the behalf of the minors/children participants involved in the study. In addition, we checked our study for compliance with the STROBE criteria [15].

We screened the database of our tertiary care centre for cardiology in Hamburg for patients ≥16 years of age, with causative *FBN1* gene mutation, 12-lead resting electrocardiography (ECG), 24-hours ambulatory ECG (AECG), and >3 months of follow-up. We did not consider patients with survival of sudden cardiac death (SCD) at baseline, neonatal Marfan syndrome [1] or known coronary artery disease [12]. Of 114 patients with *FBN1* mutations, 26 patients (23%) did not undergo AECG, and 8 patients (7%) did not have follow-up. Thus, 80 fulfilled all criteria and constituted our study group (30 men, 50 women aged 42±15 years; range 16–71 years; Table 1). Thirty-two patients were enrolled from a previous study [12]. We left all patients on medications and two expert readers who were blinded to clinical or genetic information assessed all variables on original recordings of transthoracic echocardiography (TTE), ECG, and AECG.

**Table 1:** Baseline characteristics of 80 patients with Marfan syndrome and *FBN1* mutation.

Finding	Frequency of finding*
Male	30 (38%)
Age (years)	42±15
Duration of follow-up (months)	31±18
Body weight (kg)	74±19
Body height (m)	1.84±.12
Body mass index (kg/m^2^)	21.5±4.7
Body surface area (m^2^)	1.96±.27
Marfan syndrome	76 (95%)
Sporadic Marfan syndrome	48 (60%)
Aortic root dilatation	46 (58%)
Previous aortic surgery	18 (23%)
Ectopia lentis	41 (51%)
Systemic score ≥7 points	43 (54%)

*Mean ± standard deviation or numbers (percentage).

### Diagnostic methods

**Figure 1:**
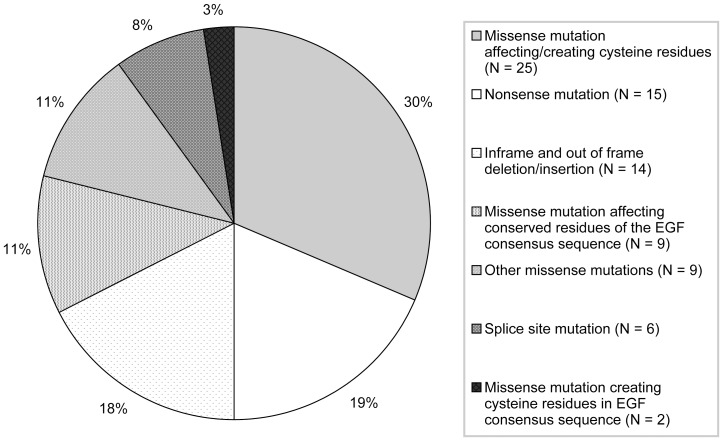
According to the revised Ghent nosology [17] we identified disease causality for 80 *FBN1* mutations, as missense mutations affecting/creating cysteine residues in 25 (30%), nonsense mutations in 15 (19%), inframe and out of frame deletion/insertions in 14 (18%), missense mutations affecting conserved residues of the EGF consensus sequence in 9 (11%), other missense mutations in 9 (11%), splice site mutations in 6 (8%), and missense mutations creating cysteine residues in a EGF consensus sequence in 2 (3%) [17].

We re-evaluated hard copies from 12-lead resting ECG as recorded on commercially available systems (CS-200, Schiller, Baar, Switzerland) with automated measurements of the resting heart rate and time intervals. All AECG recordings were performed on high-resolution, 5-channel digitized recorders (Medilog AR12, Schiller Medilog, Baar, Switzerland) with sampling rates of 4096 Hz and 16 bit accuracy, with manual pre-processing before analysis with a semi-automatic software package (Medilog Darwin Holter Analysis, Schiller Medilog, Baar, Switzerland). We recorded M-mode, two-dimensional, and colour-Doppler through optimum parasternal, apical and sub-xiphoid views using an echocardiography system (iE33, Philips Medical Systems, Eindhoven, The Netherlands) and a 4S probe [12]. We re-evaluated all measurements on an ultrasound workstation (Syngo 3.5, Siemens Medical Solutions, Erlangen, Germany). We assessed NT-proBNP serum levels at baseline with an electrochemiluminescence sandwich immunoassay (Roche Diagnostics GmbH, Mannheim, Germany) on an Elecsys System 2010 with a detection limit ≥5 pg/ml [12]. We amplified all 65 coding exons and intronic flanking splice-sites of *FBN1* (NM_000138.4) with polymerase chain reaction (PCR) from genomic deoxyribonucleic acid with previously published primers [16]. Subsequently we purified PCR products and sequenced with a Genetic Analyser (ABI 3130XL, Applied Biosystems Inc., Foster City, CA, USA). We detected gross deletions/duplications in the *FBN1* gene with multiplex ligation-dependent probe amplification (MLPA) (SALSA® MLPA® kit, probemix P065 and P066, MRC Holland, Amsterdam, Netherlands). All *FBN1* gene nucleotide changes fulfilled ≥1 Ghent criteria of causality (Figure 1) [17].

### Ventricular arrhythmia criteria

First, we assessed ventricular arrhythmia on baseline AECG [11], separately with >10 premature ventricular complexes per hour (>10 PVC/h) [11], and with ventricular couplets diagnosed with 2 sequential PVC, or with non-sustained ventricular tachycardia (nsVT) diagnosed with 4 consecutive beats to 30 seconds of arrhythmia, or both (Couplet/nsVT) [18]. Second, we assessed ventricular arrhythmia events (VTE) on follow-up which we performed by phone interviews or during clinical visits in our institution during 31±18 months (range 4–98 months). We considered VTE with SCD, sustained ventricular arrhythmia (sVT) diagnosed with regular broad-complex arrhythmia, QRS width ≥120 ms and with arrhythmia duration ≥30 seconds, with ventricular fibrillation diagnosed with grossly disorganised, rapid ventricular rhythm that varied in interval and waveform in the absences of QRS complexes [12], or with arrhythmogenic syncope, which we identified with ≥1 Sheldon score points [19]. We considered SCD only in patients with stable aortic conditions on tomographic images ≤6 months, both as witnessed cardiac arrest or death ≤1 hours after onset of symptoms, or as unexpected death with exclusion of symptoms within the previous 24 hours (Table 2) [20].

**Table 2:** Ventricular arrhythmia in 80 patients.

Finding	Number
Baseline ambulatory ECG	
- Ventricular premature beats	73 (91%)
- Ventricular premature complexes >10/h	28 (35%)
- Ventricular couplets	29 (36%)
- Bigeminy or trigeminy, or both	24 (30%)
- Non-sustained ventricular tachycardia	9 (11%)
Ventricular tachycardia events	
- Any ventricular tachycardia event*	6 (8%)
- Sustained ventricular tachycardia	4 (5%)
- Implantable cardioverter-defibrillator implantation	4 (5%)
- Sudden cardiac death	3 (4%)
- Ventricular fibrillation	2 (3%)
- Arrhythmogenic syncope	1 (1%)
- Survived resuscitation	1 (1%)

* Five patients had >1 ventricular event.

### Baseline variables

Since all patients included in this study had a causative *FBN1* gene mutation, the revised Ghent nosology required only a single additional criterion comprising aortic root dilatation, ectopia lentis, or a systemic score ≥7 points to diagnose Marfan syndrome [17], where 4 patients did not fulfil criteria of Marfan syndrome. We considered sporadic Marfan syndrome in patients who did not have a family history of Marfan syndrome and thus represented persons with sporadic *FBN1* mutations causing the condition [17], [21], aortic dilatation with diameters of the aortic sinuses ≥95th percentile of normal [22], or with aortic replacement, ectopia lentis with any displacement of the lenses [17], or after surgery for ectopia lentis, and a systemic score ≥7 points with presence of manifestations as defined by Ghent criteria [17]. We documented medication with any current intake of antiarrhythmic drugs according to the Vaughan Williams classification [23], [24], or with intake of any other medication comprising angiotensin converting enzyme inhibitors (ACE-I) and angiotensin-receptor blockers (ARB). We calculated body surface area (BSA) according to Du Bois [25]. Fasting lipid levels were available in 58 patients. We obtained systolic and diastolic blood pressures from the dominant arm after a 15-min rest on standard sphygmomanometer (Table 3).

**Table 3:** Baseline characteristics according to arrhythmia.

	Ventricular premature complexes >10/h			Ventricular couplets, or nsVT, or both			Ventricular tachycardia events		
	Absent	Present		Absent	Present		Absent	Present	
Variable	(N = 52)	(N = 28)	*P* *	(N = 48)	(N = 32)	*P* *	(N = 74)	(N = 6)	*P* *
Male gender	17 (33%)	13 (46%)	.238	18 (38%)	12 (38%)	1.000	26 (35%)	4 (67%)	.190
Age (years)	39±14	46±16	.037	39±13	45±16	.099	41±15	52±14	.070
Duration of follow-up (months)	50±37	52±33	.432	49±32	52±39	.331	51±35	40±34	.168
Body surface area (m^2^)	1.9±.08	2.01±.25	.188	1.94±.24	1.98±.31	.567	1.96±.27	1.98±.29	.289
Marfan syndrome	50 (96%)	26 (93%)	.609	46 (96%)	30 (94%)	.457	70 (95%)	6 (100%)	1.000
Sporadic Marfan syndrome	32 (62%)	16 (57%)	.812	19 (37%)	13 (41%)	1.000	46 (62%)	4 (67%)	.170
Aortic root dilatation	27 (52%)	19 (68%)	.236	26 (54%)	20 (63%)	.497	42 (57%)	4 (67%)	1.000
Previous aortic surgery	13 (25%)	5 (17%)	.580	12 (25%)	6 (19%)	.592	18 (24%)	0	.328
Ectopia lentis	27 (52%)	14 (50%)	1.000	26 (54%)	15 (47%)	.649	38 (51%)	3 (50%)	1.000
Systemic score ≥7 points	26 (50%)	17 (61%)	.481	25 (52%)	18 (56%)	.820	40 (54%)	3 (50%)	1.000
Antiarrhythmic medication									
- Class I	1 (2%)	0	1.000	1 (2%)	0	1.000	1 (4%)	0	1.000
- Class II	32 (62%)	15 (54%)	.634	26 (54%)	21 (66%)	.359	44 (60%)	3 (50%)	.687
- Class III	0	2 (7%)	.120	0	2 (6%)	.157	0	2 (33%)	.005
- Class IV	5 (10%)	3 (11%)	1.000	5 (10%)	3 (9%)	1.000	7 (10%)	1 (17%)	.480
- Any (class I–IV)†	35 (67%)	17 (61%)	.626	29 (60%)	23 (72%)	.344	49 (66%)	3 (50%)	.417
Other medication									
- ACE-I	9 (17%)	8 (29%)	.263	10 (21%)	7 (22%)	1.000	14 (19%)	3 (50%)	.107
- ARB	9 (17%)	6 (21%)	.766	6 (13%)	9 (28%)	.090	13 (18%)	2 (33%)	.313
- Any (ACE-I or ARB)	18 (34%)	14 (50%)	.233	16 (33%)	16 (50%)	.166	27 (37%)	5 (83%)	.035
Total cholesterol (mg/dl)	181±40	201±36	.143	186±41	193±38	.516	189±38	200±64	.869
HDL cholesterol (mg/dl)	64±27	59±16	.854	62±25	63±21	.634	62±23	59±27	.749
LDL cholesterol (mg/dl)	89±28	117±32	.121	96±31	108±35	.350	100±32	106±53	.933
Systolic blood pressure (mm Hg)	125±12	123±20	.430	125±12	123±19	.741	124±16	120±16	.473
Diastolic blood pressure (mm Hg)	72±10	74±10	.793	72±10	73±9	.648	73±10	75±14	.670

ACE-I identifies angiotensin converting enzyme inhibitors; ARB, angiotensin-receptor blockers; HDL cholesterol, high-density lipoprotein cholesterol; LDL, low-density lipoprotein cholesterol, and nsVT, non-sustained ventricular tachycardia.

*Mann–Whitney test for continuous data and the Fisher's exact test for nominal and categorical data.

† Three patients received two or three different classes of drugs.

### Echocardiographic variables

We assessed the left ventricular (LV) ejection fraction on apical 2- and 4-chamber views using Simpson's rule, end-systolic LV diameters, end-diastolic LV diameters, and left atrial diameters on 2-dimensional images according to current guidelines [26], with adjustment for differences in body size by dividing LV and atrial diameters by BSA [12]. We assessed aortic root diameters in patients without previous aortic surgery at the sinuses with leading edge to leading edge measurements at end-diastole in parasternal long-axis views [27], prolapse of mitral leaflets with posterior or anterior late systolic prolapse on M-mode or on 2-dimensional echocardiography from parasternal long axis views and apical 4-chamber views as leaflet displacement >2 mm [28]. We graded valve regurgitations according to current criteria; no patient exhibited severe valvular insufficiencies or previous mitral valve surgery (Table 4) [29].

**Table 4:** Clinical variables according to arrhythmia.

	Ventricular premature complexes >10/h			Ventricular couplets, or nsVT, or both			Ventricular tachycardia events		
	Absent	Present		Absent	Present		Absent	Present	
Variable	(N = 52)	(N = 28)	*P* *	(N = 48)	(N = 32)	*P* *	(N = 74)	(N = 6)	*P* *
Echocardiography									
- LV ejection fraction (%)	58±10	51±15	.058	58±9	52±15	.030	56±12	47±11	.054
- Indexed end-systolic LV diameter (mm/m^2^)	16±4	20±5	.024	17±4	20±5	.020	18±5	19±4	.241
- Indexed end-diastolic LV diameter (mm/m^2^)	27±5	29±5	.062	27±5	29±5	.029	28±5	28±4	.787
- Indexed left atrial diameter (mm/m^2^)	19±5	21±5	.039	19±5	22±5	.014	19±5	24±5	.026
- Aortic root diameter (mm)	40±10	41±7	.280	40±10	40±8	.599	40±9	40±8	.942
- Aortic valve regurgitation (moderate)	9 (17%)	8 (27%)	.263	7 (15%)	10 (31%)	.097	14 (19%)	3 (50%)	.107
- Mitral valve prolapse	29 (56%)	16 (57%)	1.000	24 (50%)	21 (66%)	.250	42 (57%)	3 (50%)	1.000
- Mitral valve regurgitation (moderate)	18 (35%)	18 (64%)	.018	15 (31%)	21 (66%)	.003	31 (42%)	5 (83%)	.085
NT-proBNP (pg/ml)	255±480	1129±2651	<.001	265±482	1032±2538	.001	342±564	3331±5345	.001
Resting ECG									
- Atrial fibrillation	3 (6%)	3 (11%)	.417	1 (2%)	5 (16%)	.035	5 (7%)	1 (17%)	.384
- Heart rate (beats/min)	68±14	71±12	.534	69±13	70±13	.589	69±14	73±5	.224
- PQ interval (ms)	158±34	171±27	.077	155±23	175±42	.017	163±32	163±35	.967
- QRS width (ms)	95±17	103±29	.147	95±23	101±22	.060	97±20	106±44	.985
- QT interval (ms)	395±32	403±39	.086	394±37	405±37	.214	398±34	401±44	.971
- QTc interval (ms)	417±32	436±29	.001	416±32	435±29	.006	422±31	442±44	.285
- Sokolow-Lyon voltage SV_1_ + RV_5_ (mV)	20±13	24±15	.195	19±10	35±17	.077	21±14	24±11	.623
- Left bundle-branch block	2 (4%)	3 (11%)	.337	1 (2%)	4 (13%)	.151	5 (7%)	0	1.000
- Right bundle-branch block	7 (14%)	5 (18%)	.744	6 (13%)	6 (19%)	.529	11 (15%)	1 (17%)	1.000
- Early repolarization	3 (6%)	2 (7%)	1.000	4 (8%)	1 (3%)	.643	5 (7%)	0	1.000
Ambulatory ECG									
- Maximal heart rate ambulatory ECG (bpm)	136±32	115±33	.009	133±33	121±34	.071	131±33	101±23	.021
- Minimal heart rate ambulatory ECG (bpm)	55±23	63±33	.135	56±25	60±31	.579	58±28	53±7	.553
- Mean heart rate ambulatory ECG (bpm)	80±12	77±11	.077	81±12	77±11	.061	80±11	70±11	.053
- Ventricular premature beats (total)	32±72	1682±2798	<.001	377±2095	957±1249	<.001	914±914	3023±5699	.144
- Ventricular couplets (total)	.3±.8	52±93	<.001	0	42±85	<.001	16±55	50±110	.376
- Ventricular bigeminy or trigeminy (total)	1.5±6.8	126±224	<.001	32±135	70±165	.006	40±125	161±337	.581
- nsVT (total)	.02±.2	1.4±4.6	.002	0	1.13±4.13	<.001	.17±.56	4.8±10	.067

LV identifies left ventricle; nsVT, non-sustained ventricular tachycardia; NT-proBNP, N-terminal pro–brain natriuretic peptide; and total, total number of findings.

*Mann–Whitney test for continuous data and the Fisher's exact test for nominal and categorical data.

### Resting ECG variables

We assessed the voltage criterion SV_1_+RV_5/6_>3.5 mV according to Sokolow and Lyon, left - and right bundle-branch blocks manually using standard criteria, and the QT intervals with correction for heart rate using the Bazett formula [12]. We documented early repolarization with a J-point elevation >0.2 mV that was either notched (with a positive J deflection inscribed on the S wave) or slurred (with smooth transition from QRS to ST-segment) in ≥2 consecutive inferior (II, III-, and aVF) or lateral (I, aVL-, and V_4_ through V_6_) leads (Table 4) [30].

### Ambulatory ECG variables

We re-evaluated all AECG recordings to assess minimal, maximal and mean heart rates, PVC, ventricular couplets, and nsVT, identified with presence of ≥3 consecutive PVC at a heart rate ≥100 beats/min. All recordings had sufficient signal quality and a minimum duration of 18 hours (Table 4) [12].

### *FBN1* mutation characteristics

To assess *FBN1* mutation characteristics we compared premature truncation codon-mutations versus inframe mutations, and splicing mutations versus all other mutations. In mutations with elimination or creation of a cysteine, with location in a calcium-binding epidermal growth factor-like (cbEGF) domain, or in a latent transforming-growth-factor beta–binding protein-like (LTBP) domain, or in exons 24–32, we first compared all mutations with the respective characteristic versus all other exon mutations, and second only missense mutations with the respective characteristic versus all other missense mutations (Table 5) [1], [31].

**Table 5:** *FBN1* mutation characteristics according to arrhythmia.

	Ventricular premature complexes >10/h			Ventricular couplets, or nsVT, or both			Ventricular tachycardia events		
	Absent	Present		Absent	Present		Absent	Present	
Variable	(N = 52)	(N = 28)	*P* *	(N = 48)	(N = 32)	*P* *	(N = 74)	(N = 6)	*P* *
Premature truncation codon mutations	14 (27%)	11 (39%)	.314	14 (29%)	11 (34%)	.632	21/74 (28%)	4/6 (67%)	.073
Splicing mutations	2 (4%)	4 (14%)	.176	4 (8%)	2 (6%)	1.000	6/74 (8%)	0/6	1.000
Mutation affecting cysteine	22/50 (44%)	5/24 (21%)	.072	18/44 (41%)	9/30 (30%)	.461	26/68 (38%)	1/6 (17%)	.406
Missense mutation affecting cysteine	22/32 (69%)	5/13 (39%)	.094	18/27(67%)	9/18 (50%)	.355	26/42 (62%)	1/3 (33%)	.555
Mutation in cbEGF domain	5/50 (10%)	2/24 (8%)	1.000	4/44 (9%)	3/30 (10%)	1.000	6/68 (9%)	1/6 (17%)	.461
Missense mutation in cbEGF domain	5/50 (10%)	2/11 (18%)	1.000	4/28 (14%)	3/16 (19%)	.692	6/42 (14%)	1/2 (50%)	.296
Mutation in LTBP domain	1/50 (2%)	5/24 (21%)	.012	0/44	6/30 (20%)	.003	5/68 (7%)	1/6 (17%)	.409
Missense mutation in LTBP domain	0/36	3/13 (23%)	.016	0/30	3/19 (16%)	.053	7/47 (6%)	0	1.000
Mutation in exons 24–32	5/50 (10%)	4/24 (17%)	.460	4/44 (9%)	5/30 (17%)	.471	6/68 (9%)	3 (50%)	.021
Missense mutation in exons 24–32	3/32 (9%)	3/11 (27%)	.164	2/27 (7%)	4/16 (25%)	.174	5/41 (12%)	1/2 (50%)	.262

*Mann–Whitney test for continuous data and the Fisher's exact test for nominal and categorical data.

### Data analysis

**Figure 2:**
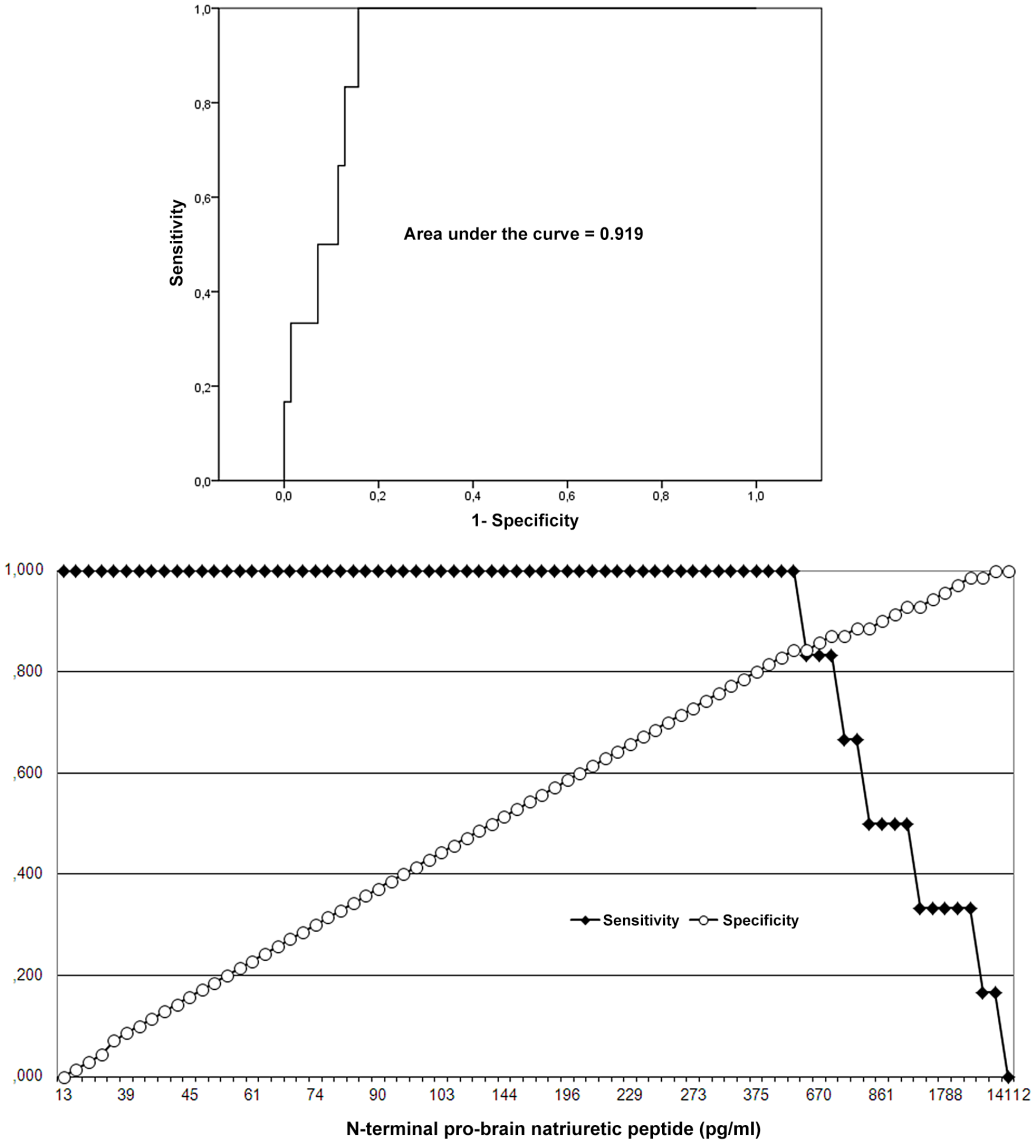
ROC curve analysis identifies NT-proBNP serum levels >618 pg/ml as a threshold for increased risk of VTE. The area under the curve is .919 (95% confidence interval .850 to.988; *P*<.001; upper panel). For better identification of cut-offs separating high and low risk, we separately display sensitivity and specificity (lower panel).

**Figure 3:**
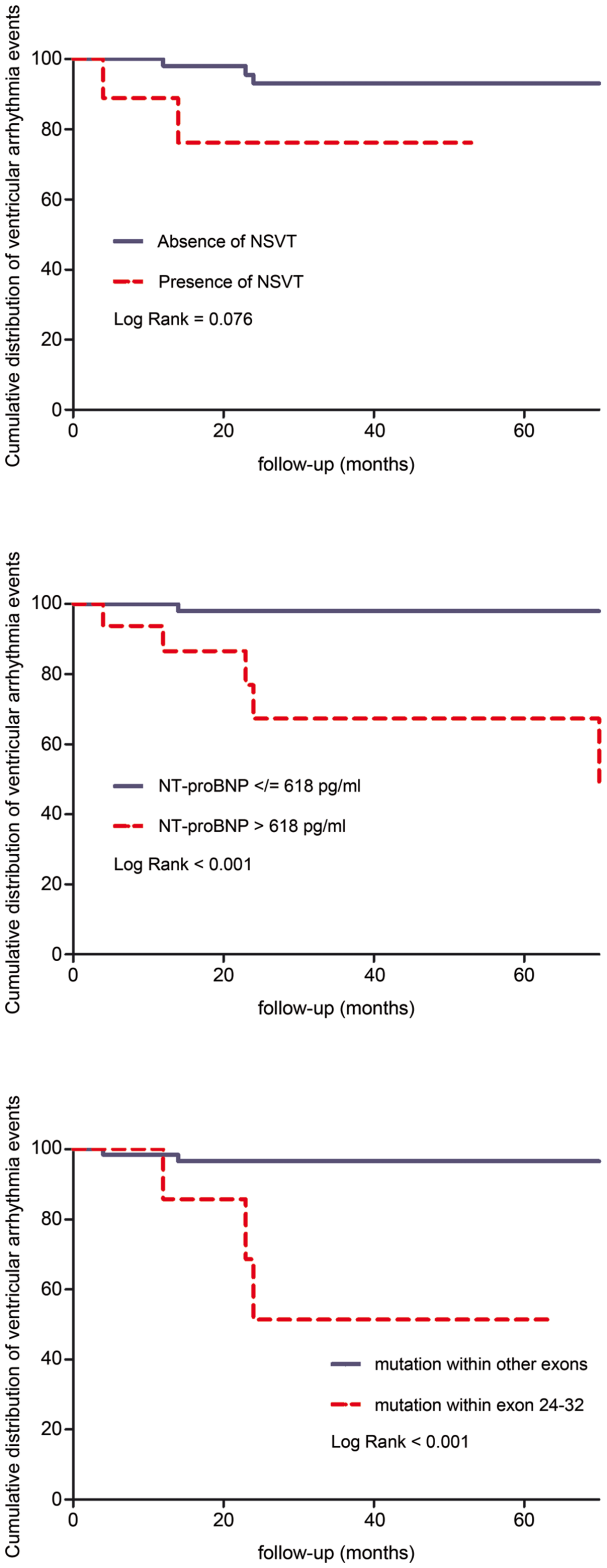
Kaplan–Meier curves indicate an increased cumulative risk for VTE depending on presence of nsVT (upper panel), of NT-proBNP serum levels >618 pg/ml (middle panel), and of *FBN1* gene mutation in exons 24–32 (lower panel).

We performed exploratory comparisons of variables with the Mann–Whitney test for continuous data and the Fisher's exact test for nominal data (Tables 3, 4, 5). Receiver operating characteristic (ROC) curve analysis was used to identify NT-proBNP serum level thresholds with increased risk of VTE (Figure 2). Kaplan-Meier analysis displayed cumulative event-free functions with the Log rank to screen for differences (Figure 3). All *P*-values were two-sided, where we considered *P*-values <.05 statistically meaningful. There was no missing data, and we expressed quantitative data as means ± standard deviation and qualitative data as numbers (percentage). We used a standard software package (PASW Statistics for Windows, Release 18.0.0, SPSS Inc. 2009, Chicago, Illinois) for all statistical tests.

## Results

We identified >10 PVC/h in 28 (35%), Couplet/nsVT in 32 (40%), and VTE in 6 of 80 (8%) patients with *FBN1* mutations, including 3 with SCD (4%). Two SCD happened despite implantation of a cardioverter-defibrillator (ICD) for sVT, and one SCD after arrhythmogenic syncopes. The other 3 patients with VTE exhibited sVT with recommendation of an ICD, with refusal in one (Table 2). With the exception of medication, baseline characteristics were similar irrespective of ventricular arrhythmia (Table 3).

PVC>10/h, Couplet/nsVT, and VTE exhibited increased NT-proBNP (all *P*<.001) and increased indexed left-atrial diameters (all *P*<.05). PVC>10/h and Couplet/nsVT related to increased indexed end-systolic LV diameters (*P* = .024 and *P* = .020), to moderate mitral valve regurgitation (*P* = .018 and *P* = .003), and to prolonged QTc intervals (*P* = .001 and *P* = .006), respectively. In addition, PVC>10/h related to decreased LV ejection fractions (*P* = .030), and to prolonged PQ-intervals (*P* = .017). Finally, maximal heart rates were decreased in PVC>10/h (*P* = .009) and in VTE (*P* = .021; Table 4).

Mutations located in LTBP domains, of both all types and only missense mutations were associated with PVC>10/h (*P* = .012 and *P* = .016) and with Couplets/nsVT (*P* = .003 and *P* = .053), respectively. Most notably, VTE was significantly more common in patients with mutations in exons 24–32 (*P* = .021; Table 5). ROC curve analysis identified NT-proBNP levels >618 pg/ml as threshold for increased risk of VTE (Figure 2). Kaplan–Meier analysis corroborated an association of VTE with increased NT-poBNP (*P*<.001) and with mutations in exons 24–32 (*P*<.001), but documented only marginal association with nsVT (*P* = .076; Figure 3). There was no significant cut-off for an increased risk of VTE depending on the number of PVC, or nsVT (data not shown).

## Discussion

The study shows that adults with *FBN1* gene mutations have a high prevalence both, of ventricular arrhythmia on baseline AECG (48%) and of VTE (8%) including SCD (4%) during follow-up. Moreover, myocardial dysfunction and location of mutations in exons 24–32 of the *FBN1* gene emerge as risk factors for VTE.

The frequency of 8% for VTE including 4% with SCD is much higher than in the general population [12] but lower than in hypertrophic cardiomyopathy, where 12% of patients experience SCD [32]. Autopsy was not available, but we applied stringent criteria for SCD, and other VTE preceded all SCDs. Five of our 6 patients with VTE had sVT with recommendation of ICD implantation, and a single patient without sVT had arrhythmogenic syncope with ≥50 PVC/h and nsVT prior to SCD. Thus, VTE was well-documented in all 6 patients.

The diagnostic utility of AECG in Marfan patients requires elucidation. Yetman et al reported 3 patients with SCD in a total of 70 patients with Marfan syndrome and long-term follow-up, and all 3 of their patients with SCD exhibited PVC>10/h on AECG [11]. Similarly, in the study of 77 patients with Marfan syndrome by Hoffman et al, the number of PVC on AECG was significantly higher in those 7 patients with VTE [12]. Moreover, PVC>10/h are also documented to predict VTE after myocardial infarction [18], and nsVT relate to SCD in congestive heart failure [18]. However, in our current study we could not establish any single criterion on AECG that clearly identified an increased risk for VTE. We also failed to identify a cut-off for an increased risk of VTE depending on the number of PVC, or nsVT. On the other hand, with the exception of a single individual all patients with VTE exhibited ≥1 sign of ventricular arrhythmia. Thus, we feel that AECG does not provide any our-right criteria that qualify as a diagnostic test to safely identify or to exclude a risk for SCD in patients with Marfan syndrome. However, patients with Marfan syndrome who exhibit PVC>10/h, or ventricular couplets, or nsVT may warrant closer clinical monitoring especially when other risk factors may also be present.

Both, ventricular arrhythmia and VTE related to increased NT-proBNP levels, increased LV diameters, decreased LV ejection fractions, or moderate mitral valve regurgitation. Thus myocardial dysfunction emerges as a risk for ventricular arrhythmia in *FBN1* mutations. Previous studies of Marfan patients also identified myocardial dysfunction as a risk factor of ventricular arrhythmia [9]–[12]. Myocardial dysfunction may be multifactorial including primary myocardial impairment, hemodynamic relevant valve regurgitation with myocardial stretch, increased aortic wall stiffness, and sleep apnea [12], [33]. Interestingly, our threshold of NT-proBNP levels >618 pg/ml for an increased risk of VTE is higher than the NT-proBNP level threshold ≥214.3 pg/ml identified by Hoffmann et al [12]. However, both studies are based on small numbers of patients and thresholds may be tested in larger populations prior to generalization as a diagnostic test.

Our finding of lower maximal heart rates and prolonged PQ- and QTc intervals in PVC>10/h and in Couplet/nsVT is also described in clinically diagnosed Marfan syndrome [10]–[12], where primary tissue defects due to *FBN1* gene mutations may be causative. Of note, 5 patients with *FBN1* mutation exhibited early repolarization, which was unrelated with ventricular arrhythmia [30].

We found that medication with angiotensin converting enzyme inhibitors, angiotensin-receptor blockers, and class III antiarrhythmic drug were more frequent in the group of patients with VTE. However, these medications were unlikely to increase the risk of VTE themselves [5], but their prescription might rather be seen as a response to the perception of an increased cardiovascular risk in these patients.

Our distribution of *FBN1* mutation classes was comparable with findings in 1.013 *FBN1* mutations [1]. Most notably, we observed association of VTE with mutations in exons 24–32. Mutations in these exons carry a well-known risk for aortic complications, mitral valve abnormalities, and reduced survival [1]. Our data suggest that reduced survival in these mutations may also relate to SCD. The serious effect of these mutations was previously explained by location in the central stretch of contiguous EGF-like domains and by overrepresentation of missense mutations [1]. However, in our study only 5 of 9 mutations in exons 24–32 were missense mutations. Finally, PVC>10/h and Couplet/nsVT were more prevalent in mutations in LTBP domains. These mutations also increase the risk for mitral valve surgery [34], which underpins the potential relationship of mitral valve regurgitation and ventricular arrhythmia.

### Study limitations

Potential limits of our study need discussion. First, our patients with heritable connective tissue disease adequately represent the frequency of these diseases in the Hamburg metropolitan population [35]. However, in the current study only 80 of 114 patients with *FBN1* mutations fulfilled inclusion criteria, where 26 patients (23%) were excluded because they did not undergo AECG. However, we recommend AECG to all patients with Marfan-like disorders irrespective suspicion or known arrhythmia, and the majority of patients who did not undergo this 24-hour investigation wished to avoid numerous visits at our tertiary care centre. Thus, we do not believe that there was a substantial bias for patients receiving AECG with higher risk for arrhythmia. Second, with a number of events per variable of less than 10, multiple testing results get biased [36]. Thus, given the low number of events in our study patients we were unable to establish an independent impact of variables on VTE. Most importantly, it remains to be clarified, whether arrhythmia may be viewed as a primary consequence of *FBN1* mutation characteristics itself, or whether arrhythmia is a result from secondary conditions such as myocardial ischemia, mitral valve abnormality, or ventricular dysfunction. Consequently, clinical and molecular risk factors of ventricular arrhythmia require further investigation in large multi-centre trials of patients with *FBN1* mutations using advanced monitoring technology such as loop recorders. Finally, we included 4 patients with a causative *FBN1* mutation who remained below current diagnostic thresholds for the final diagnosis of Marfan syndrome. However, these individuals had a family history of Marfan syndrome, and their phenotype was not suggestive of alternative syndromes such as isolated kyphoscoliosis, MASS phenotype, ectopia lentis, familial thoracic ascending aortic aneurysms and dissections, Shprintzen-Goldberg syndrome, Weill-Marchesani syndrome, stiff skin syndrome or acromicric and geleophysic dysplasia [1].

## Conclusions

Marfan syndrome with causative *FBN1* mutations is associated with an increased risk for arrhythmia, and affected persons may require life-long monitoring. Ventricular arrhythmia on electrocardiography, signs of myocardial dysfunction and mutations in exons 24–32 may be risk factors of VTE. Large multi-centre trials should investigate risk factors and preventive and therapeutic options for *FBN1* gene-related ventricular arrhythmia.
